# Mechanisms and Clinical Consequences of Vascular Calcification

**DOI:** 10.3389/fendo.2012.00095

**Published:** 2012-08-06

**Authors:** Dongxing Zhu, Neil C. W. Mackenzie, Colin Farquharson, Vicky E. MacRae

**Affiliations:** ^1^The Roslin Institute and Royal (Dick) School of Veterinary Studies, The University of EdinburghMidlothian, Scotland, UK

**Keywords:** vascular calcification, vascular smooth muscle cells, therapeutic strategies

## Abstract

Vascular calcification has severe clinical consequences and is considered an accurate predictor of future adverse cardiovascular events, including myocardial infarction and stroke. Previously vascular calcification was thought to be a passive process which involved the deposition of calcium and phosphate in arteries and cardiac valves. However, recent studies have shown that vascular calcification is a highly regulated, cell-mediated process similar to bone formation. In this article, we outline the current understanding of key mechanisms governing vascular calcification and highlight the clinical consequences. By understanding better the molecular pathways and genetic circuitry responsible for the pathological mineralization process novel drug targets may be identified and exploited to combat and reduce the detrimental effects of vascular calcification on human health.

## Introduction

Whilst it is tempting to presume that vascular calcification is a product of modern society, this pathological process was actually first documented in an autopsy of the mummy of an elderly Egyptian woman, which revealed calcific aortic atherosclerosis (Czermack, 1852). Following this report, Johann Georg Mönckeberg described medial calcific sclerosis, a form of arteriosclerosis or vessel hardening, where calcium deposits are found in the muscular middle layer of the walls of arteries (Mönckeberg, 1903). For many years, vascular calcification was regarded as a passive and degenerative disease without treatment options (Virchow, 1989). Over the past two decades, extensive research has conclusively shown that pathological vascular calcification is a tightly regulated process that shares many similarities with physiological bone mineralization (Shanahan et al., 1999; Moe and Chen, 2004; Vattikuti and Towler, 2004; Hruska et al., 2005; Demer and Tintut, 2008; Sage et al., 2011; Zhu et al., 2011). However, the precise mechanisms through which vascular calcification occurs still remains unclear. Progressively enlarging calcium deposits are seen in the major arteries of individuals older than 60 years of age (Allison et al., 2004). Extensive vascular calcification is also frequently observed in patients with atherosclerosis, chronic kidney disease (CKD), and diabetes (Fuchs et al., 1985; Sangiorgi et al., 1998; Allison et al., 2004; Giachelli, 2004; Reaven and Sacks, 2005; Okuno et al., 2007; Shroff and Shanahan, 2007; Kestenbaum et al., 2009). The incidence of vascular calcification is highly correlated with mortality and morbidity of cardiovascular disease (Arad et al., 2000; Rosenhek et al., 2000; Keelan et al., 2001; Wayhs et al., 2002), in the form of reduced aortic compliance, decreased cardiac efficiency, deterioration of coronary perfusion, and subendocardial ischemia (Kelly et al., 1992; Watanabe et al., 1992; Ohtsuka et al., 1994). Therefore the identification and characterization of novel mediators of vascular calcification will offer the potential for future therapeutics to inhibit progression or induce regression of vascular calcification. In this review, we will describe the mechanisms underpinning vascular calcification and discuss the risk factors, clinical consequences, and potential therapeutic targets for this pathological process. Abbreviations and acronyms are detailed in Table 1.

**Table 1:** **Abbreviations and acronyms**.

Abbreviations and acronyms	
ANK	Ankylosis protein
CAVS	Calcific aortic valve stenosis
CKD	Chronic kidney disease
CVC	Calcifying vascular cell
ESRD	End stage renal disease
GACI	Generalized arterial calcification of infancy
MGP	Matrix Gla protein
NPP1	Ecto-nucleotide pyrophosphatase/phosphodiesterases-1
NSAID	Non-steroidal anti-inflammatory drug
OPG	Osteoprotegerin
OPN	Osteopontin
Pi	Inorganic phosphate
PPi	Inorganic pyrophosphate
TNAP	Tissue non-specific alkaline phosphatase
VKDP	Vitamin K-dependent protein
VSMC	Vascular smooth muscle cell

### Types of vascular calcification

Vascular calcification can be categorized into four main types according to location: atherosclerotic intimal calcification, medial artery calcification (Mönckeberg’s sclerosis), cardiac valve calcification, and calcific uremic arteriolopathy. Histologically, calcified deposits may be amorphic, chondromorphic, or osteomorphic in structure, and may be characterized as metastatic or dystrophic.

#### Atheroslcerotic intimal calcification

Atherosclerosis is the development of plaques within the intimal layer of large vessels, and underlies coronary artery disease and cerebrovascular disease, the most common forms of life threatening cardiovascular disorders (Doherty et al., 2004). Atherosclerosis can by induced by chronic inflammation and lipid deposition, with dyslipidemia frequently linked to the severity of calcium deposition (Pohle et al., 2001; Schmermund et al., 2001). Atherosclerotic calcification is the most common form of calcific vasculopathy, and occurs as early as the second decade of life just after fatty streak formation (Stary et al., 1995). Small aggregates of crystalline calcium can be detected in developing lesions, and in adults past the fourth decade of life, greater lesion areas may be calcified (Stary, 2000). The degree of calcification correlates with the extent of atherosclerosis, with age, and hypertension as dominant risk factors for systemic calcified atherosclerosis (Allison et al., 2004). The predominant mineral form in calcified lesions is hydroxyapatite which may initially form in membrane bound matrix vesicles that bud from the membranes of chondrocytes and osteoblasts present within deposits of cartilage and bone tissue, respectively (Yu, 1974). In addition to mineral, these lesions also contain matrix vesicles as well as outright bone and cartilage (Tanimura et al., 1983; Mohler et al., 2001; Hunt et al., 2002).

#### Medial calcification

Medial calcification, also termed Mönckeberg’s sclerosis, occurs in the tunica media of blood vessels. It is a characteristic feature of Generalized Arterial Calcification of Infancy (GACI), diabetes, and CKD (Fuchs et al., 1985; Chen and Moe, 2003; Rutsch et al., 2003, 2008; London et al., 2005; Reaven and Sacks, 2005; Okuno et al., 2007; Kestenbaum et al., 2009), and is associated with increased cardiovascular mortality and amputation risk (Chantelau et al., 1995; Lehto et al., 1996; London et al., 2003). Medial calcification occurs independently of atherosclerotic calcification and is a process similar to intramembranous bone formation, with no cartilaginous precursor required (Towler et al., 1998; Vattikuti and Towler, 2004). Calcium deposition can be observed throughout most of the medial width in the early stage of disease. At later stages of disease, the media is filled with circumferential rings of mineral. In some cases, osteocytes and bone trabeculae can also be observed (Shanahan et al., 1999; Zhu et al., 2011).

#### Cardiac valve calcification

**Figure 1:**
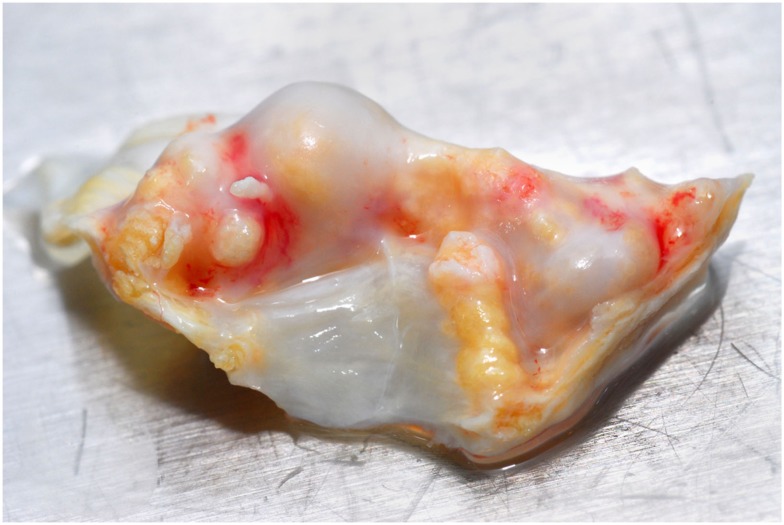
**Extensive calcific aortic valve stenosis in the valve leaflets of a patient undergoing valve replacement surgery**.

Heart valves allow unidirectional blood flow through the heart. The four main valves of the mammalian heart are: the two atrioventricular (AV) valves and the two semilunar (SL) valves. The AV valves including the mitral valve and the tricuspid valve are located between the atria and the ventricles. The SL valves are the aortic valve and the pulmonary valve and are located in the arteries leaving the heart. Calcific aortic valve disease is identified by thickening and calcification of the aortic valve leaflets (Figure 1) in the absence of rheumatic heart disease. It is divided into aortic sclerosis, in which the leaflets do not obstruct left ventricular outflow, and aortic stenosis, in which obstruction to the left ventricular outflow is present. A number of recent studies have shown that calcific aortic valve lesions have many features characteristic of an actively cell-regulated process, including lipoprotein deposition (O’Brien et al., 1996; Olsson et al., 1999), chronic inflammation (Olsson et al., 1994; Otto et al., 1994), and active calcification (Mohler et al., 2001; Rajamannan et al., 2003) and shares similar underlying mechanisms with atherosclerotic intimal calcification (Salhiyyah et al., 2011).

#### Calcific uremic arteriolopathy

Calcific uremic arteriolopathy is a severe type of widespread medial vascular calcification which occurs in blood vessels of patients with End stage renal disease (ESRD) often referred to as stage 5 CKD (Qunibi et al., 2002). It affects cutaneous and subcutaneous arteries and arterioles, leading to intimal proliferation, fibrosis, and thrombosis (Qunibi et al., 2002; Mwipatayi et al., 2007).

### The cellular sources of vascular calcification

Cells that spontaneously produce calcified matrix and undergo a bone-like transdifferentiation include vascular smooth muscle cells (VSMCs), pericytes, and calcifying vascular cells (CVCs). These cell types are closely related and may be variant phenotypes of one another (Minasi et al., 2002; Tintut et al., 2003).

#### Vascular smooth muscle cells

**Figure 2:**
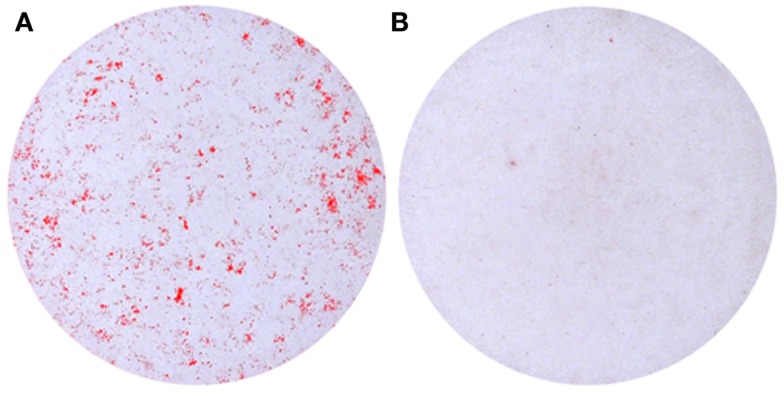
**Calcification of murine vascular smooth muscle cells cultured *in vitro* in the presence of (A) high phosphate (2.5 mM) compared to (B) control medium**.

Vascular smooth muscle cells normally reside in the media of blood vessels and are responsible for regulating vascular tone. VSMCs exhibit a contractile phenotype and highly express genes which are required for the maintenance of myofilament structure and function. These genes include α-smooth muscle-actin (SMA), SM22α, and SM-myosin heavy chain (Shanahan et al., 1993; Mackenzie et al., 2011). VSMCs can be activated from a quiescent, differentiated state into an actively proliferating and synthesizing phenotype (Hedin et al., 1999). This phenotypic change is associated with loss of smooth muscle cell markers and can be induced by various stimuli *in vitro*, including various growth factors, injury, or mechanical stress (Thyberg, 1996; Worth et al., 2001). VSMCs are thought to be the predominant cells associated with medial calcification (Essalihi et al., 2004; Narisawa et al., 2007; Zhu et al., 2011), in contrast to intimal calcification, which also involves lipids and inflammatory cells (Pohle et al., 2001; Schmermund et al., 2001). Vascular calcification is prevalent in patients with CKD, especially those with ESRD. ESRD patients typically have hyperphosphatemia compared to healthy control patients (Giachelli, 2009). A number of studies have shown that VSMCs cultured with high phosphate can undergo calcification *in vitro* (Figure 2) which involves the phenotypic transition to osteoblastic, chondrocytic, and osteocytic cells (Steitz et al., 2001; Johnson et al., 2005; Speer et al., 2009; Sage et al., 2011; Zavaczki et al., 2011; Zhu et al., 2011). This *in vitro* model has been widely used for investigating the cellular and molecular mechanisms responsible for vascular calcification.

#### Pericytes

Pericytes are elongated, contractile cells found wrapped about precapillary arterioles outside the basement membrane and are present in veins, arteries, and capillaries. Several pericyte markers have been identified, including SMA, non-muscle actin, non-muscle and muscle myosin, amino peptidase-N, amino peptidase-A, and a cell surface ganglioside (3G5; Andreeva et al., 1998). Previous studies have shown that pericytes can differentiate into osteoblasts and chondrocytes (Doherty and Canfield, 1999; Farrington-Rock et al., 2004), suggesting that pericytes may be central to the etiology of vascular calcification. Further studies have shown that signaling through the Wnt/beta-catenin pathway stimulates chondrogenic and inhibits adipogenic differentiation of pericytes (Kirton et al., 2007), which may directly contribute to the development and progression of calcium deposition. In addition, pericytes can form multicellular nodules that contain a mineralized matrix, similar to those found in calcified aortae (Doherty and Canfield, 1999; Cola et al., 2004). Molecules associated with bone development and formation have been observed in these mineralized nodules, emphasizing the regulatory similarities between vascular and bone calcification (Doherty et al., 1998; Canfield et al., 2000).

#### Calcifying vascular cells

Calcifying vascular cells are a subpopulation of smooth muscle cells which exhibit osteoblastic characteristics and undergo spontaneous calcification *in vitro* (Watson et al., 1994; Balica et al., 1997; Tintut et al., 1998; Radcliff et al., 2005). CVCs have features in common with pericytes including a similar morphology, osteoblastic characteristics, and 3G5 expression (Watson et al., 1994). During osteogenic differentiation, CVCs accumulate not only minerals but also lipids such as triglycerides. Indeed, the induction of *de novo* lipogenesis promotes the calcification of CVCs under pro-osteogenic conditions such as high phosphate levels (Ting et al., 2011). Studies characterizing the calcific nodules produced by CVCs in ApoE-null mice have revealed that the nodules resemble calcific atherosclerotic plaque and can be destabilized in the presence of active lipids and monocytes (Li et al., 2012), providing a novel animal model of vulnerable plaque dynamics.

### Valve interstitial cells

Calcification of the aortic valve occurs following trans-differentiation of the valve interstitial cells (VICs) through a myofibroblast stage into osteoblast-like cells (Liu et al., 2007). VICs are present in all three layers of the aortic valve and can be induced to differentiate into myofibroblasts by inflammatory response (often caused by endothelial damage; Liu et al., 2007) and the release of Angiotensin, TGF-β, and matrix metalloproteinases (Zhou et al., 1996;Kaden et al., 2003, 2005). After further accumulation of lipids, changes in structure, and fibrosis, differentiation to an osteoblast phenotype is thought to occur via wnt3-Lrp5-β and osteoprotegerin (OPG)/receptor Activator of Nuclear Factor Kappa B (RANK) mediated signaling pathways (Cosmi et al., 2002; Osman et al., 2006; Rajamannan, 2009). Osteoblastic cells then mediate deposition of mineral by processes associated with bone formation (Rajamannan et al., 2003).

### Mechanisms of vascular calcification

**Figure 3:**
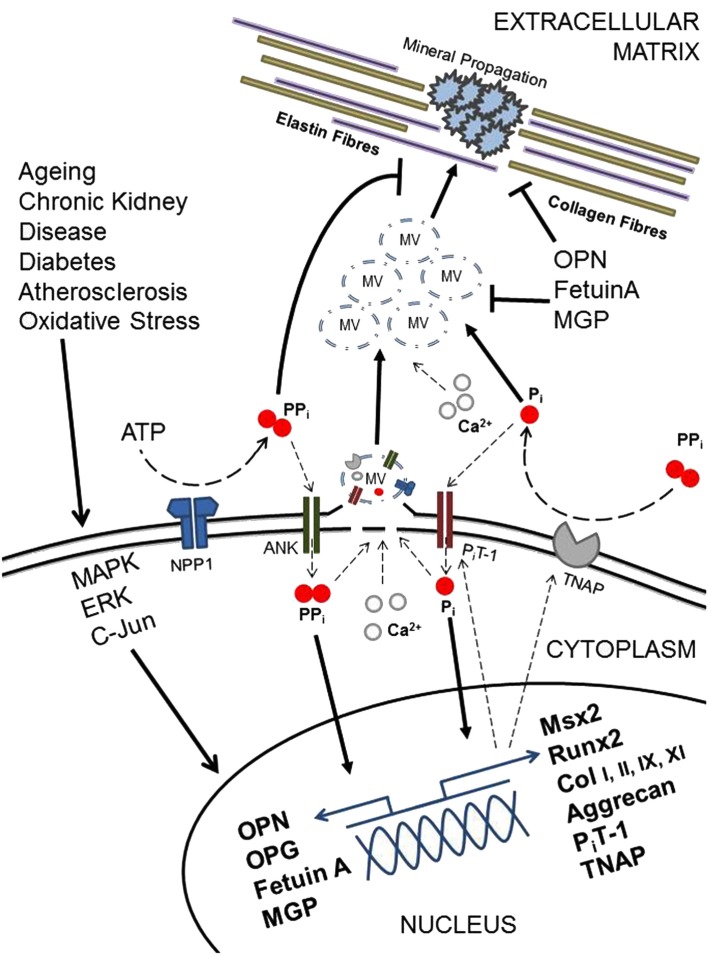
**Diagramatic representation of selected regulatory factors and their potential roles in vascular calcification**.

A series of clinical and basic science studies performed in the last several years underscored the biological complexity of the processes driving vascular calcification (Figure 3).

**Figure 4:**
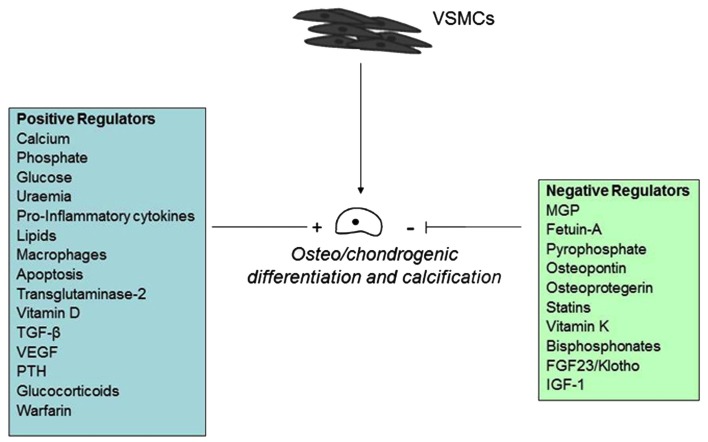
**Positive and negative regulators of vascular calcification**.

The pathological cell-mediated process of soft tissue calcification shares many similarities with that of the physiological matrix mineralization during skeletal development. Membrane-bound matrix vesicles nucleate hydroxyapatite crystals that contain calcium and inorganic phosphate (Anderson et al., 1990; Nahar et al., 2008) forming the first nidus for calcification. This occurs via a tightly controlled balance of inhibitors and inducers, including metabolic alterations (Chen et al., 2006; Kapustin et al., 2011; Sage et al., 2011; Sevinc Ok et al., 2012), inflammation (Tintut et al., 2000; Stompór et al., 2003; Lencel et al., 2011), drugs (Kirton et al., 2006; Helas et al., 2009; Beazley et al., 2012), and morphogens (Radcliff et al., 2005; Nakahara et al., 2010; Shimizu et al., 2011; Figure 4).

Matrix vesicles contain negative regulators of hydroxyapatite crystal nucleation and growth, such as fetuin-A and matrix gla protein (MGP; Reynolds et al., 2004; Murshed et al., 2005). In cooperation with local mediators such as pyrophosphate (PPi), these molecules protect the arteries from deposition and growth of minerals (Luo et al., 1997; Harmey et al., 2004; Jahnen-Dechent et al., 2011). In the absence of these inhibitors, or following the stimulation of cell death-related processes, together with the bone-like activity of vascular cells, calcification is readily induced (Canfield et al., 2002; Speer et al., 2002; Shroff et al., 2008).

Osteoblasts and chondrocytes are responsible for bone and cartilage formation and calcification within the skeleton. Normal VSMC populations contain cells that undergo phenotypic transition to osteocytic, osteoblastic, and chondrocytic cells in a calcified environment (Steitz et al., 2001; Johnson et al., 2005; Speer et al., 2009; Zhu et al., 2011). Chondro-osseous and calcification promoting genes reported in calcifying VSMCs include the transcription factor Msx2 which promotes osteoblastogenesis (Shao et al., 2005), the osteoblast master transcription factor Runx2 (Speer et al., 2009), the chondrocyte specific extracellular matrix constituent aggrecan and collagen Types I, II, IX, and XI (Johnson et al., 2008). The phosphate transporter PiT-1 is the predominant sodium-dependent phosphate co-transporter expressed in human VSMCs. Phosphate increases PiT-1 expression, which leads to increased levels of intracellular phosphate. This induces Runx2 expression and the osteogenic conversion of VSMCs (Li et al., 2006).

**Figure 5:**
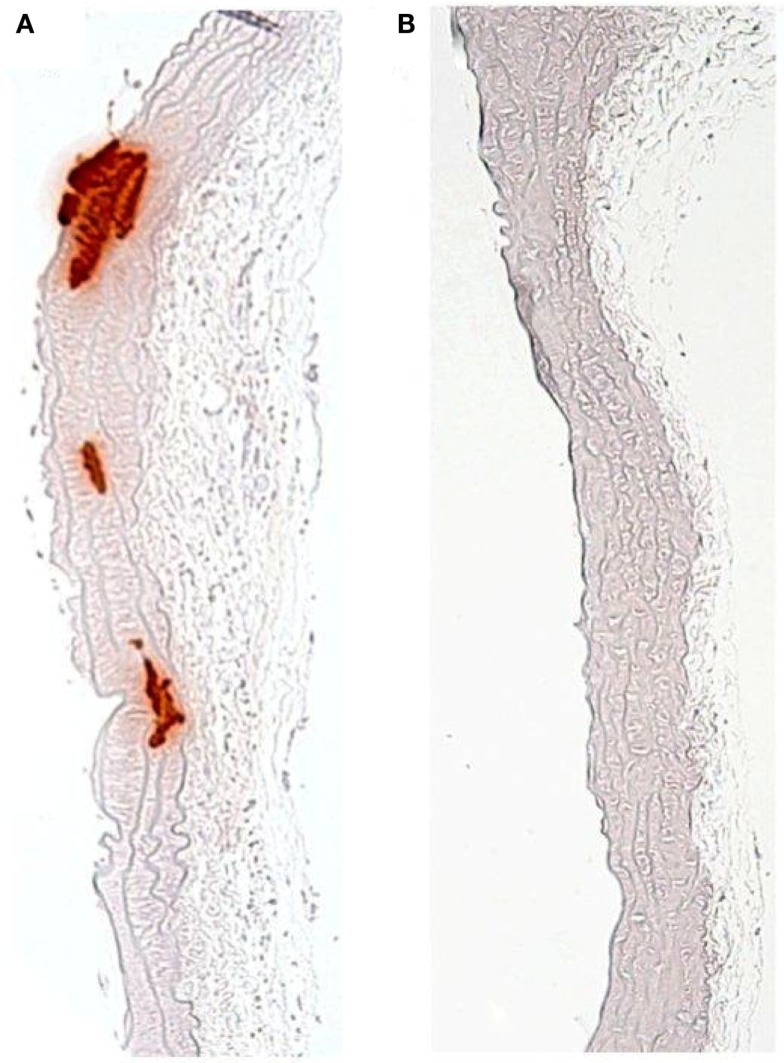
**Medial calcification of the aorta due to depressed levels of the calcification inhibitor pyrophosphate in the (A) Enpp1-null mouse, compared to (B) wild-type control**.

Tissue non-specific alkaline phosphatase (TNAP), a key enzyme for bone calcification, is also central to vascular calcification through the hydrolysis of the calcification inhibitor PPi and the generation of phosphate for hydroxyapatite formation in VSMCs (Narisawa et al., 2007; Lomashvili et al., 2008). Conversely, the ankylosis protein (ANK) and ecto-nucleotide pyrophosphatase/phosphodiesterases-1 (NPP1) inhibit vascular calcification through the promotion of extracellular PPi levels in VSMCs (Johnson et al., 2005; Narisawa et al., 2007), with mice lacking NPP1 developing severe aortic calcification (Figure 5). PPi inhibits calcium phosphate crystal growth and helps to prevent VSMC chondro-osseous differentiation and calcification (Rutsch et al., 2003; Johnson et al., 2005). Arterial calcification is also physiologically limited by VSMC expression of OPN, which is a recognized inhibitor of hydroxyapatite crystal formation and growth and promotes mineral resorption (Speer et al., 2002). OPG, the endogenous inhibitor MGP and the circulating inhibitor fetuin-A have also been shown to block VSMC calcification (Bucay et al., 1998; Canfield et al., 2002; Speer et al., 2002; Bennett et al., 2006; Matsui et al., 2009).

Changes to several intracellular signal transduction pathways have been reported during vascular calcification, including the induction of the extracellular signal-regulated kinase 1/2, c-Jun N-terminal kinase, and p38 Mitogen Activated Protein Kinase pathways (Simmons et al., 2004; Tanikawa et al., 2009). The growth arrest-specific gene 6 (Gas6)/Axl survival signal, which exerts an antiapoptotic effect through the Bcl2-mediated phosphatidylinositol 3-kinase/protein kinase b pathway has also been shown to be one of the key mechanisms for phosphate-induced calcification (Collett et al., 2007; Son et al., 2007). These studies suggest that therapeutics targeting the Axl receptor may open up new avenues for the prevention of vascular calcification *in vivo*.

## Clinical Consequences of Vascular Calcification

Calcification of blood vessels is a common consequence of aging, atherosclerosis, CKD, and diabetes (Fuchs et al., 1985; Sangiorgi et al., 1998; Allison et al., 2004; Giachelli, 2004; Reaven and Sacks, 2005; Okuno et al., 2007; Shroff and Shanahan, 2007; Kestenbaum et al., 2009; Mackenzie and MacRae, 2011) and is associated with significant mortality and morbidity of cardiovascular disease (Arad et al., 2000; Rosenhek et al., 2000; Keelan et al., 2001; Wayhs et al., 2002). Indeed clinically, vascular calcification is now accepted as a valuable predictor of coronary heart disease (Greenland et al., 2007). The clinical ramifications of vascular calcification in CKD, atherosclerosis, and cardiac valve calcification are described here in more detail.

### 

#### Chronic kidney disease

It has been reported that approximately 40% of patients with CKD have vascular calcification compared with 13% of control patients with normal renal function (Russo et al., 2004). Kramer et al. (2005) demonstrated a positive association between the presence of vascular calcification and renal failure, and that this association increased markedly in CKD diabetic patients. Converging evidence from clinical, epidemiological, and translational research studies has suggested that vascular calcification progresses inexorably during dialysis and may only partially reverse after successful transplantation (Ossareh, 2011; Shroff, 2011). Medial calcification leads to vascular stiffness and decreases the compliance of blood vessels. These changes result in both increased pulse pressure (Dao et al., 2005) and left ventricular hypertrophy (Speer and Giachelli, 2004). In dialysis patients, medial calcification contributes to calcific uremic arteriolopathy, a necrotizing skin condition with high mortality rates (Coates et al., 1998).

#### Generalized arterial calcification of infancy

Generalized Arterial Calcification of Infancy is a rare autosomal recessive disease which is characterized by the calcification of arteries, in conjunction with arterial stenosis caused by intimal proliferation. The majority of affected children die within the first 6 months of life as the result of end-organ damage. In a subset of patients, peri-articular calcification of joints also occurs (Rutsch et al., 2003, 2008).

#### Atherosclerosis

Previous studies have shown that intimal calcification is positively correlated with atherosclerotic plaque burden (Rumberger et al., 1995; Sangiorgi et al., 1998), increased risk of myocardial infarction (Beadenkopf et al., 1964; Loecker et al., 1992), and plaque instability (Fitzgerald et al., 1992; Burke et al., 2000). Furthermore, calcium deposits may directly alter atherosclerotic plaque stability (Wong et al., 2012). However, a major limitation of using calcium score progression as a marker of risk is that the positive predictive value appears to be low with substantial overlap among those with and without future adverse cardiovascular events (Greenland et al., 2007).

#### Cardiac valve calcification

In the aortic valve, calcification gives rise to life-threatening stenosis. Calcific aortic valve stenosis (CAVS) is the leading reason for valve replacement in Europe and North America, and is considered to be a major mode of failure of native as well as bioprosthetic valves (O’Keefe et al., 1991; Lindroos et al., 1993). CAVS is also correlated with a high risk of cardiovascular dysfunction, and is the third leading cause of cardiovascular disease (Ribeiro et al., 1998; Nkomo et al., 2006).

### Risk factors for vascular calcification

Elevated serum phosphate levels are recognized as a major risk factor for cardiovascular events in the general population (Dhingra et al., 2007; Kestenbaum et al., 2009) and in CKD (Young et al., 2005; Adeney et al., 2009). Serum phosphate levels greater than 5.5 mg/dL are strongly correlated with mortality in ESRD patients (Block et al., 2004; Tentori et al., 2008). Furthermore, relatively small increases in serum phosphate (3.5–4.5 mg/dL) have also been correlated with increased risk of cardiovascular and all-cause mortality in CKD patients (Kestenbaum et al., 2005) and the general population with normal renal function (Tonelli et al., 2005). Increased susceptibility of CKD patients to vascular calcification likely underlies this high risk of cardiovascular disease-related deaths in CKD patients.

A number of clinical studies have also shown an association between elevated serum calcium and increased risk of myocardial infarction and vascular calcification in both CKD patients and in the general population (Yamada et al., 2007; Kovesdy et al., 2010; Larsson et al., 2010; West et al., 2010). Furthermore, a recent meta-analysis has reported that dietary calcium supplementation is associated with a significantly increased risk of myocardial infarction (Bolland et al., 2010). Clinical studies investigating the patterns of systemic atherosclerotic calcification have further revealed age and hypertension as the dominant risk factors for calcification (Allison et al., 2004).

In recent years, several studies have demonstrated the positive relationship between vascular calcification and bone health (Frye et al., 1992; Kiel et al., 2001). Vascular calcification is often accompanied by either decreased bone mineral density or disturbed bone turnover. This association has been observed in general populations (Hyder et al., 2007) and also in patients with osteoporosis, Paget’s disease, and CKD (Laroche and Delmotte, 2005; Raggi et al., 2007; Toussaint et al., 2008; Osako et al., 2010; Bandeira et al., 2012). It appears that in patients with CKD that both extremes of bone remodeling, low turnover (adynamic bone), and hyperparathyroid bone, may accelerate vascular calcification by not allowing calcium or phosphorus into bone, or resorbing it out of bone, respectively (Moe and Chen, 2004). In genetically altered animals with deletions of OPG and klotho, a combined osteoporosis-arterial calcification phenotype has been observed (Bucay et al., 1998; Nabeshima, 2002). Furthermore, bone loss and vascular calcification share various common mechanisms, including estrogen deficiency, vitamin D and K abnormalities, chronic inflammation, and oxidative stress (Hofbauer et al., 2007).

Calcific aortic valve stenosis is associated with classic atherosclerotic risk factors, including hypercholesterolemia, hypertension, smoking, and male gender (Mohler et al., 1991; Stewart et al., 1997). A faster disease progression has also been reported in patients with a metabolic syndrome (Briand et al., 2006). Lifestyle modifications are therefore likely to be advantageous, however a beneficial effect of controlling cardiovascular risk factors has yet to be demonstrated in CAVS.

### Potential therapeutic targets

Specific drugs capable of inhibiting vascular calcification have yet to be developed. Potential strategies that have recently been investigated include the administration of vitamin K, statins, bisphosphonates, TNAP inhibitors, and Non-Steroidal Anti-Inflammatory Drugs (NSAIDs).

### Vitamin K

The vitamin K-dependent proteins (VKDPs) MGP and Gas-6 are produced by VSMCs and pericytes. The process of converting VKDPs to their biologically active forms requires the carboxylation of glutamic acid residues by vitamin K (Furie et al., 1999). In rats, inactivation of MGP by treatment with the vitamin K antagonist warfarin leads to rapid calcification of the arteries. This can be regressed by a vitamin K-rich diet (Schurgers et al., 2007). Specifically, Vitamin K2 supplementation prevents arterial calcification, yet vitamin K1 does not (Howe and Webster, 2000; Spronk et al., 2003). In the population based Rotterdam study, increased intake of vitamin K2, but not K1, was shown to be inversely related to all-cause mortality (relative risk = 0.91) and severe aortic calcification (relative risk = 0.74; Geleijnse et al., 2004). A more recent investigation examined the association of vitamin K1 and vitamin K2 intake with coronary calcification in a cross-sectional study among 564 post-menopausal women (Beulens et al., 2009) 0.62% of the women had coronary calcification. Vitamin K2 intake was again associated with decreased coronary calcification (relative risk = 0.80). Interestingly, one of the major dietary sources of vitamin K2 is cheese (Schurgers and Vermeer, 2000), which although is not related to a healthy lifestyle or diet, has yet to be established as a dietary risk factor for cardiovascular disease. It is therefore possible that cheese could exert a beneficial effect in the cardiovascular system and that the high cheese consumption in France and the Mediterranean countries may possibly account for the lower prevalence of cardiovascular disease.

#### Statins

The mechanism attributed to the pleiotrophic effects of statins involves the inhibition of RhoA/Rho-kinase (ROCK) activity (Rikitake and Liao, 2005). Inhibition of ROCK with the inhibitor Y-27632 or siRNA significantly increased ALP activity and calcification of bovine VSMCs and rat aorta organ cultures (Chen et al., 2010). Furthermore, matrix vesicles isolated from bovine VSMCs incubated with Y-27632, show increased ALP activity and increased ability of MVs to subsequently calcify collagen by 66% (Chen et al., 2010). Together these data clearly demonstrate that the RhoA/ROCK signaling pathway is an important negative regulator of vascular calcification. Exposure to fluvastatin has been shown to directly inhibit calcification in VSMCs *in vitro*, with warfarin treatment abolishing this beneficial effect (Nakano-Kurimoto et al., 2009). Atorvastatin has also been shown to protect cultured VSMCs from phosphate-induced calcification by inhibiting apoptosis via restoration of the Gas6-Axl pathway (Son et al., 2007). However, the clinical use of statins has yet to be shown to effectively inhibit vascular calcification, with neither fulvastatin (Forbat et al., 1998) nor atorvastatin (Schmermund et al., 2006) therapy able to attenuate coronary artery calcification progression. Furthermore a recent clinical trial focusing on changes in coronary artery plaque composition and plaque volume during aggressive dual lipid-lowering therapy with atorvastatin and ezetimibe demonstrated no significant differences in plaque calcification (Kovarnik et al., 2012). These clinical data may be due to statins inhibiting the initiation rather than the progression of vascular calcification.

#### Bisphosphonates

Bisphosphonates are used as standard therapy for osteoporosis. Studies in rats have shown that alendronate and ibandronate inhibit warfarin and uremia induced media calcification at doses that inhibit bone resorption (Price et al., 2001, 2006). However, it has recently been reported that whilst etidronate and pamidronate prevent the development of vascular calcification in rats with adenine-induced chronic renal failure, bone formation, and mineralization are adversely affected (Lomashvili et al., 2009). These findings support and extend previous results showing that the most effective etidronate dose for the prevention of arterial calcification also reduced bone mineral density in 5/6-nephrectomized rats (Tamura et al., 2007). In 2008, a multicenter genetic study and retrospective observational analysis of subjects affected by GACI revealed a positive association between survival and bisphosphonate treatment (Rutsch et al., 2008). More recently, the long-term survival of a severe case of GACI diagnosed prenatally and treated with etidronate over a 2-year period has been reported. Progressive resolution of arterial calcification was seen by 3 months of age, which was maintained until 2 years of age. Throughout the 2-year follow-up the patient developed mild hypophosphatemia, due to renal phosphate wasting, without signs of rickets (Edouard et al., 2011). This study supports the development of a formalized approach for the treatment of GACI with bisphosphonates.

#### TNAP inhibitors

Novel TNAP inhibitors which result in higher PPi levels and lower Pi levels have been reported (Narisawa et al., 2007). These compounds have been shown to be capable of reducing *in vitro* VSMC calcification, and will serve as scaffolds for future efforts to develop novel drugs for the treatment of soft tissue calcification.

#### Non-steroidal anti-inflammatory drugs

Non-Steroidal Anti-Inflammatory Drugs are commonly used for anti-inflammation and analgesia post-operatively in orthopedic patients. However, several studies have demonstrated that these drugs suppress bone growth, remodeling, and repair (Nilsson et al., 1986; Keller et al., 1987; Ho et al., 1995) through mechanisms including cell cycle arrest and cell death induction (Chang et al., 2005). The administration of Tanshinone IIA, one of the major lipophilic components extracted from the root of *Salvia miltiorrhiza Bunge* (Shang et al., 2012), attenuates atherosclerotic calcification in a rat model, through inhibition of oxidative stress (Tang et al., 2007). Furthermore, the natural antioxidants curcumin and silybin inhibit VSMC calcification *in vitro* (Roman-Garcia et al., 2011). However, the cyclooxygenase-2 inhibitor Celecoxib induced no significant changes in atherosclerotic calcification in a mouse model of atherosclerosis (Bea et al., 2003). Further studies are therefore required to more fully investigate the potential therapeutic applications of NSAIDs in suppressing vascular calcification.

## Conclusion

Vascular calcification is associated with a number of human diseases including CKD, diabetes, and atherosclerosis, and is a significant independent risk factor for the development of cardiovascular disease. Whilst the molecular mechanisms of vascular calcification are similar to the process of bone mineralization, further insights are required to determine the precise pathways involved that will allow the identification of effective targets for the development of novel therapeutics.

## Conflict of Interest Statement

The authors declare that the research was conducted in the absence of any commercial or financial relationships that could be construed as a potential conflict of interest.
